# Cylindroma of the neck in a male patient: a case report

**DOI:** 10.1186/s13256-025-05566-8

**Published:** 2025-10-30

**Authors:** Omar Alwakaa, Sawsan Abo Saada, Hasan Eid, Aladdin Etr

**Affiliations:** 1 Harvard Medical School, Beth Israel Deaconess Medical Center, Boston, MA USA; 2 Faculty of Medicine, University of Aleppo, Aleppo, Aleppo Governorate Syrian Arab Republic; 3 Department of Plastic Surgery, Aleppo University Hospital, Aleppo University, Aleppo, Aleppo Governorate Syrian Arab Republic

**Keywords:** Cylindroma, Skin neoplasms, Neck neoplasms, Brooke–Spiegler syndrome, Dermatopathology, Oncology, Surgery

## Abstract

**Background:**

Cylindroma is a benign adnexal tumor mostly found on the scalp or face. They present as pink to purple, smooth-surfaced, solitary or multiple nodular lesions, mostly affecting middle-aged and elderly women. Reports of solitary cylindromas occurring in other anatomical locations are rare.

**Case presentation:**

We present a unique case of a 56-year-old Arab male patient with a solitary, large, non-ulcerated cylindroma on the left-sided neck. The lesion had progressively enlarged since childhood, with worsening symptoms such as pain and a burning sensation over the last 2 years. A second, smaller cystic lesion was found behind the left ear. The patient had no significant family or past medical history, and specifically no history of skin diseases. Surgical excision of both lesions was performed. Histopathology confirmed a benign dermal cylindroma and a keratinous epidermal cyst. The lesion demonstrated characteristic histological features: irregular basaloid islands in a jigsaw pattern surrounded by hyaline basement membranes. Postoperative recovery was uneventful.

**Conclusion:**

This case highlights a rare clinical presentation of a large benign cylindroma in an unusual location. Surgical excision remains an effective and definitive treatment.

## Introduction

Cylindroma is a rare benign adnexal tumor characterized as a slow-growing lesion, most often observed on the face or scalp. It is histologically characterized by basaloid cell islands arranged in a jigsaw pattern. In some cases, multiple cutaneous cylindromas could grow on the scalp until they coalesce together and form a single large mass that resembles headwear, such as a turban or hat [1]. Malignant transformation and locally aggressive behavior are rare, and when they occur, they are generally associated with long-standing scalp turban tumors [2]. Moreover, metastatic malignant cylindromas are extremely rare as well. Generally, cylindroma affects women more frequently than men, with a reported female-to-male ratio ranging from 6:1 to 9:1, typically presenting between the ages of 30 and 50 years [3]. Cylindroma is classified into three primary subtypes: cutaneous lesions; salivary gland lesions known as adenoid cystic carcinoma, which are malignant; and multiple lesions associated with familial history such as Brooke–Spiegler syndrome (BSS), which is an autosomal dominant condition involving multiple adnexal tumors, exhibiting a higher potential for malignant transformation compared with isolated cutaneous lesions. Although most cases are diagnosed between the third and fifth decades of life, some lesions may remain indolent for years before demonstrating accelerated growth. Historically, several hereditary disorders, including multiple familial trichoepitheliomas, BSS, and familial cylindromatosis, have been considered separate entities. However, recent genetic research has revealed that these disorders are related to each other, as they all involve heterogeneous mutations at the same gene locus (CYLD) [4]. This case presents a unique solitary neck cylindroma in a male patient. It expands the known clinical spectrum of this neoplasm and emphasizes the importance of surgical excision as curative treatment. To our knowledge, such a presentation has not been widely reported in Syria or the Middle East [5, 6].

## Case report presentation

**Fig. 1:**
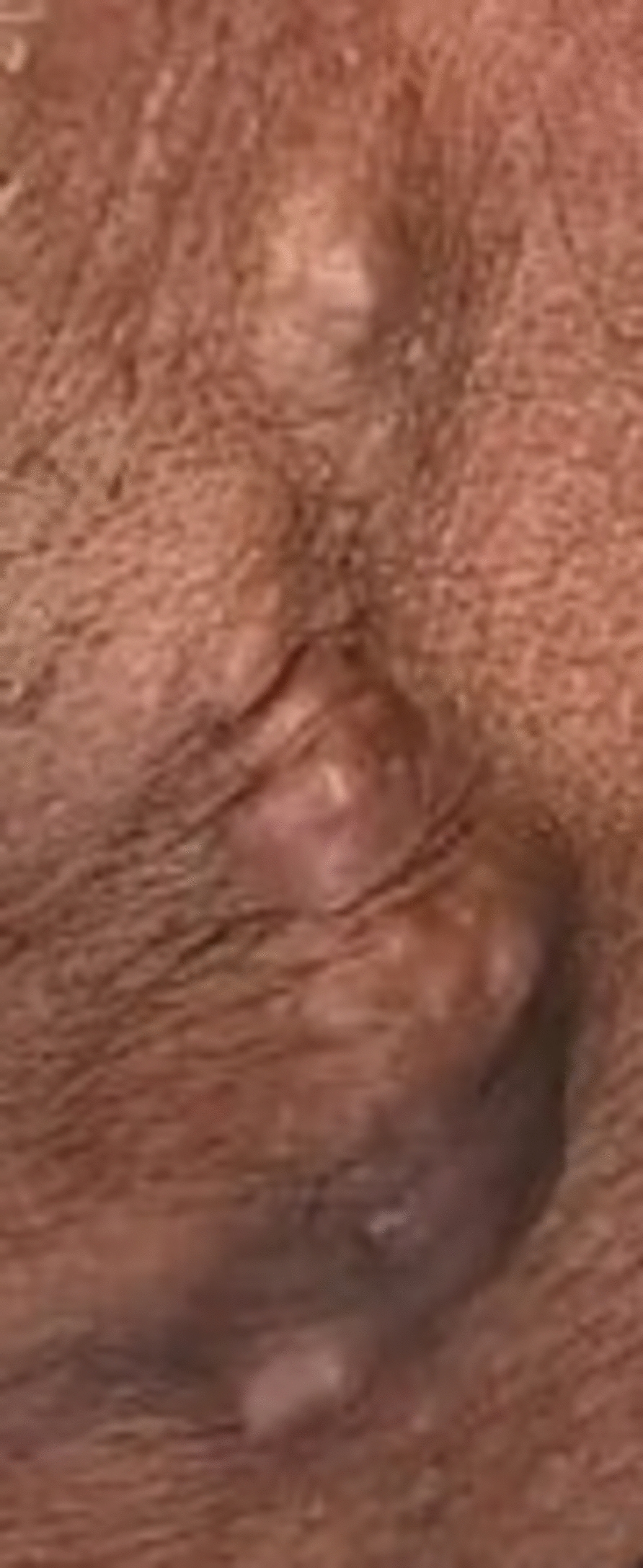
Lateral view of the patient’s neck showing the skin lesion on the left-sided neck

A 56-year-old Arab Syrian man, smoker with no significant medical history, presented to the plastic surgery department for a skin lesion on the left-sided neck that had been present since the patient was 7 years old. However, the lesion experienced a gradual increase in size, became more painful, and caused burning sensation over the last 2 years. On examination, the mass was firm, raised, skin colored, free movable, and measured 10.5 × 3 cm (Fig. 1). The lesion was accompanied by the presence of a small cystic structure behind the left ear measuring 2.6 × 2 cm. No other lesions were found on his scalp and there were no abnormalities in other areas of the skin, mucosa, nails, or hair. There were no signs of ulceration, fistula, inflammation, or active infection. The patient also denied any family history of similar skin lesions.

**Fig. 2:**
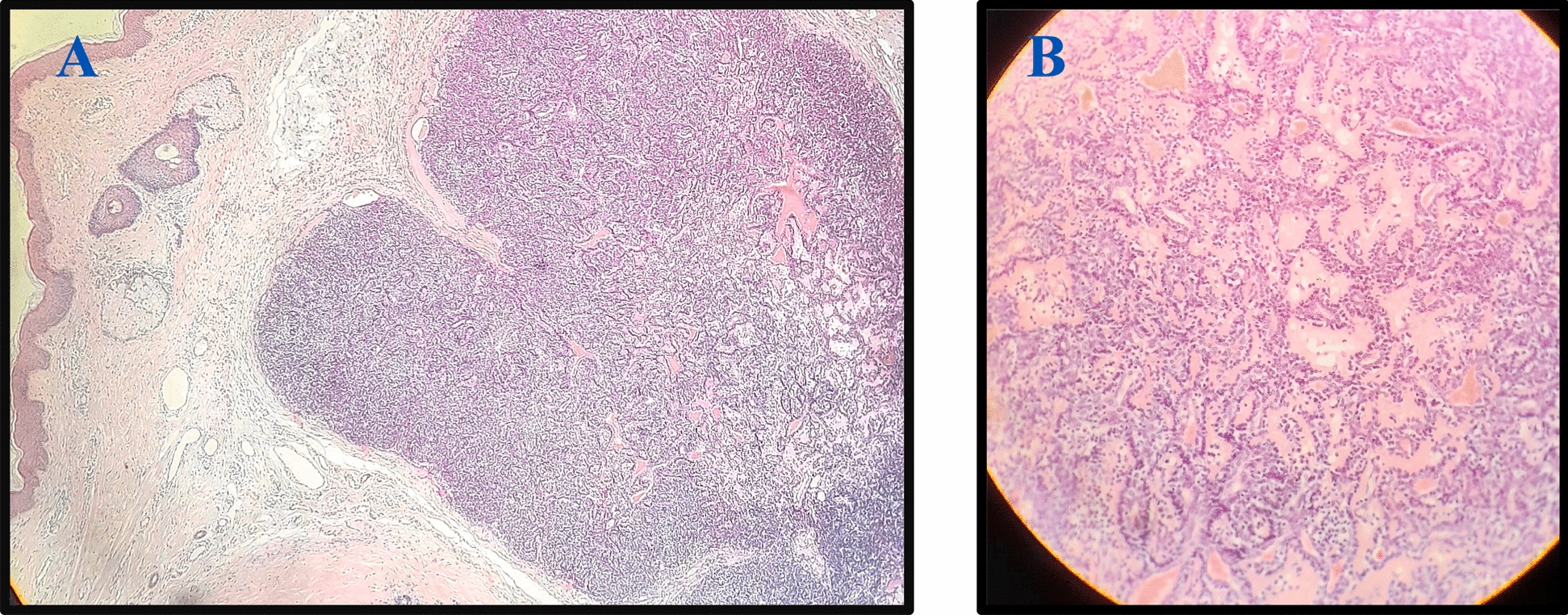
Microscopic view of the excised lesion confirming the diagnosis of cylindroma. **A** Encapsulated subcutaneous tumor with compact nests of basaloid cells arranged together resembling a jigsaw puzzle pattern. **B** Histopathological section demonstrates “jigsaw”-like nests infiltrating into the dermis and subcutaneous tissues. These irregular nests of basaloid cells are surrounded by a dense hyaline basement membrane

**Fig. 3:**
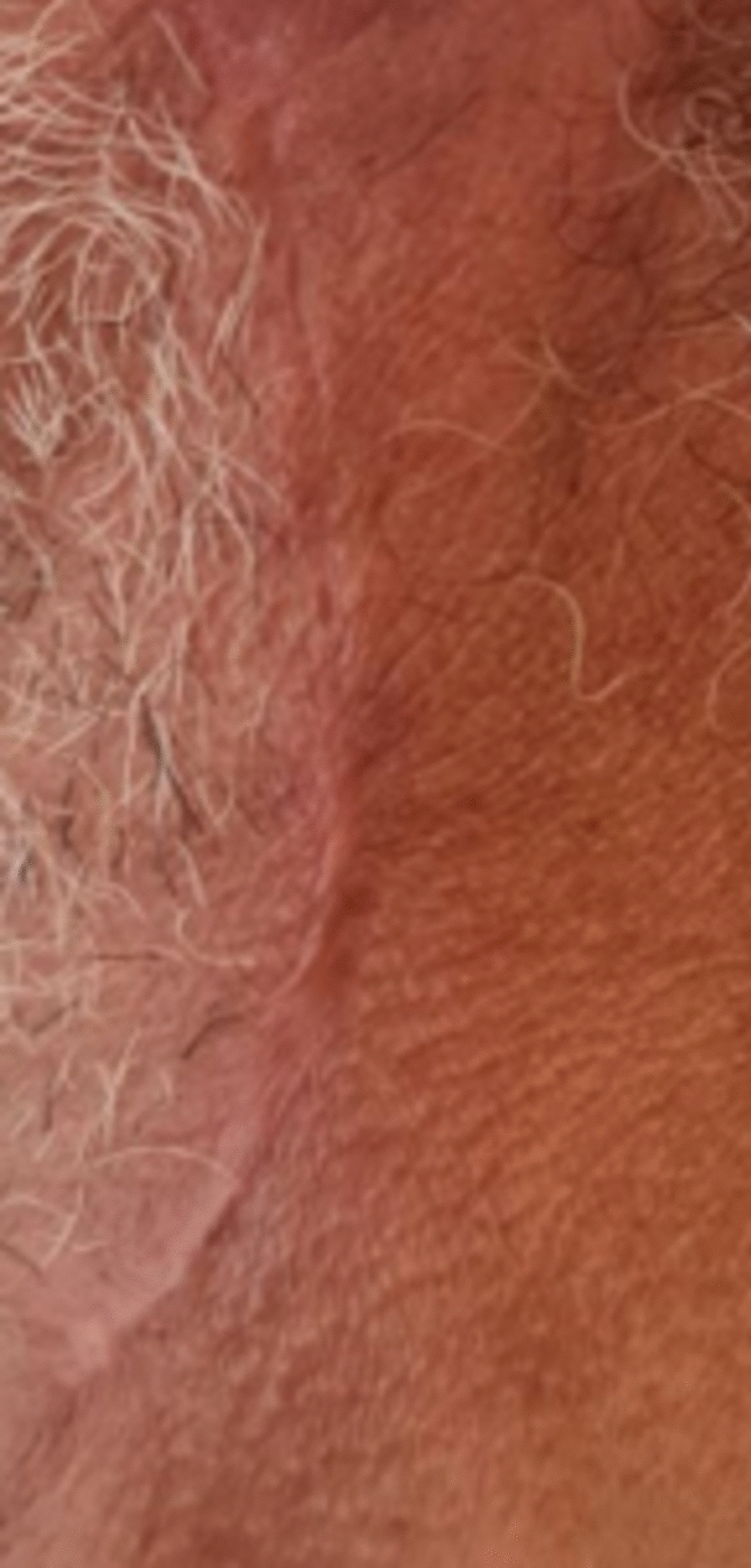
Lateral view of the patient’s neck showing post-surgical healing along the incision line, with no signs of infection or inflammation. The surgical scar is visible below the earlobe, indicating the location of the previous intervention

The patient underwent surgical excision of the skin lesion and the cystic structure. Primary closure of the wound was performed under general anesthesia. The excised specimens were sent for histopathological examination. There were no signs of fistula or communication with other nearby structures. Histopathologic examination of the resected skin tissue revealed many small irregular islands of basaloid cells in the dermis surrounded by thick hyaline basement membrane with some small hyaline droplets within a few islands. The irregular islands of the basaloid cells were connected to each other as a compact nest or as a “jigsaw”-like puzzle (Fig. 2). The final histopathological report diagnosed the mass as “benign dermal cylindroma of the left-sided neck.” The histopathological examination of the resected cystic structure showed normal epidermis, with one small dermal cystic structure lined by keratinized stratified squamous epithelium. The cyst was filled with keratin flakes and surrounded by chronic inflammatory cells. Healing of the wound and recovery of the patient were both uneventful. On last clinical follow‑up 2 months post-procedure, the wound was completely healed, and the patient was doing well without any clinical complaints (Fig. 3).

## Discussion

Patients with cylindroma can present with a red linear plaque of tiny superficial nodules on the scalp or neck, or pre-auricular nodules. These lesions can grow gradually over time or be present from birth [1]. The presented case is unique due to the presence of a non-ulcerated cylindroma located on the left-sided neck, accompanied by an epidermal cyst behind the left ear. Additionally, the unusually large size of the lesion further distinguishes this case. Moreover, the presented case is notable due to the lesion’s long duration and sudden progression. The patient reported noticing the lesion since early childhood but reported a gradual increase in lesion size over the last 2 years. The delayed rapid growth and onset of pain may be attributed to age-related hormonal changes, minor unnoticed trauma, or internal cystic degeneration. These mechanisms have been hypothesized in the literature as triggers for dormant adnexal tumors becoming symptomatic in adulthood [7–9].

Histopathological examination of benign cylindroma is characterized by mosaic nests of undifferentiated basaloid cells with small, dark nuclei and scant cytoplasm [10]. The microscopic view of the skin lesion specimen in our case showed these nests, which are arranged in a distinctive “jigsaw puzzle” pattern, with larger nodules fitting together neatly and firmly [11]. It also showed that these nests are typically surrounded by a thick layer of PAS-Periodic Acid-Schiff stain and highly eosinophilic basement membrane. The immunoprofile of cylindromas is similar to that of other benign appendageal neoplasms. They are usually identified through an inflammatory component composed of cytokeratin-expressing cells, Langerhans cells, and T-lymphocytes dispersed throughout the tumor tissue [12].

Although several strategies have been investigated for cylindroma treatment, surgical excision has been reported to be the preferred approach for isolated lesions [13]. As alternate surgical treatment, cryotherapy and electrodesiccation with curettage could also be an option. Furthermore, minor cylindroma lesions have been effectively treated using carbon dioxide laser [14]. Although progressive removal of patient’s nodules over time might reduce the need for more invasive surgical treatments, patients with several cylindromas may require plastic surgery. Most importantly, surgical management is often indicated due to the deformity caused by the tumor and its potential to impact quality of life. Surgical treatment could offer a better cosmetic outcome and could reduce the risk of malignant progression. In general, it is considered a safe and effective treatment, with a favorable recovery profile and minimal postoperative discomfort [15].

It is important to mention that the patient did not report a family history of similar lesions, and only one cylindroma was identified on histopathology. Although the presence of an epidermal cyst behind the ear raised a minor suspicion for BSS, the lack of multiple adnexal tumors or histological evidence of trichoepithelioma or spiradenoma supports a diagnosis of sporadic cylindroma. Nevertheless, BSS is known to exhibit variable penetrance, and absence of family history does not exclude the diagnosis. Genetic testing for CYLD mutations, which is the definitive test, was not performed due to lack of access [16]. CYLD is a gene that can prevent tumorigenesis by inhibiting nuclear-factor-kappa-beta (NF-κB) activation. Loss of CYLD function is associated with tumor growth and dysregulated tropomyosin kinase (TRK) signaling in CYLD cutaneous syndrome [17]. Therefore, it has been proposed that nonsurgical therapy, such as the use of NF-κB inhibitors, might postpone or prevent surgery in cylinroma treatment. In addition, TRK inhibitors could also represent a treatment option for tumors that lack functional CYLD, such as cylindroma [18]. Furthermore, long-term surveillance and regular follow-up are recommended for patients with multiple cylindromas due to likelihood of high recurrence and risk of recurrence or malignant transformation [19]. In our case, we treated our patient surgically because the skin lesion was a symptomatic solitary large lesion. Moreover, we wanted to avoid the side effects of nonsurgical therapies, such as immunodeficiencies and hepatotoxicity through the use of NF-κB inhibitors [20].

Our case report disproves the existing hypothesis that cylindroma is exclusively congenital in origin by demonstrating that they can develop at a later age. Unfortunately, a comprehensive evaluation of the lesion’s pathogenesis has not been obtained. Due to specific economical and logistical challenges, genetic testing for CYLD mutations could not be conducted in our case. This fact could be considered as a limitation of our case report. Nonetheless, this case contributes valuable insights into the clinical and histopathological spectrum of cylindromas and emphasizes the importance of ongoing cylindroma research.

## Conclusion

Our case report illustrates a rare presentation of a large benign cylindroma on the neck as an atypical location, successfully treated with surgical excision. The large size of the lesion and progressive symptoms, including pain and burning sensation, emphasize the importance of early recognition and intervention in similar cases. Surgical excision proved to be an effective and safe treatment.

## Data Availability

The datasets during and/or analyzed during the current study available from the corresponding author on reasonable request.
